# Axonal mapping of the motor cranial nerves

**DOI:** 10.3389/fnana.2023.1198042

**Published:** 2023-06-02

**Authors:** Vlad Tereshenko, Udo Maierhofer, Dominik C. Dotzauer, Gregor Laengle, Olga Politikou, Genova Carrero Rojas, Christopher Festin, Matthias Luft, Florian J. Jaklin, Laura A. Hruby, Andreas Gohritz, Dario Farina, Roland Blumer, Konstantin D. Bergmeister, Oskar C. Aszmann

**Affiliations:** ^1^Clinical Laboratory for Bionic Extremity Reconstruction, Department of Plastic, Reconstructive and Aesthetic Surgery, Medical University of Vienna, Vienna, Austria; ^2^Division of Plastic and Reconstructive Surgery, Massachusetts General Hospital, Harvard Medical School, Boston, MA, United States; ^3^Center for Anatomy and Cell Biology, Medical University of Vienna, Vienna, Austria; ^4^Department of Plastic, Aesthetic and Reconstructive Surgery, University Hospital St. Pölten, Karl Landsteiner University of Health Sciences, Krems an der Donau, Austria; ^5^Department of Orthopedics and Trauma Surgery, Medical University of Vienna, Vienna, Austria; ^6^Department of Plastic Surgery, University of Basel, Basel, Switzerland; ^7^Department of Bioengineering, Imperial College London, London, United Kingdom; ^8^Department of Plastic, Reconstructive and Aesthetic Surgery, Medical University of Vienna, Vienna, Austria

**Keywords:** facial nerve, hypoglossal nerve, masseteric nerve, facial muscles, sensory feedback, sympathetic axons, motor control, proprioception

## Abstract

Basic behaviors, such as swallowing, speech, and emotional expressions are the result of a highly coordinated interplay between multiple muscles of the head. Control mechanisms of such highly tuned movements remain poorly understood. Here, we investigated the neural components responsible for motor control of the facial, masticatory, and tongue muscles in humans using specific molecular markers (ChAT, MBP, NF, TH). Our findings showed that a higher number of motor axonal population is responsible for facial expressions and tongue movements, compared to muscles in the upper extremity. Sensory axons appear to be responsible for neural feedback from cutaneous mechanoreceptors to control the movement of facial muscles and the tongue. The newly discovered sympathetic axonal population in the facial nerve is hypothesized to be responsible for involuntary control of the muscle tone. These findings shed light on the pivotal role of high efferent input and rich somatosensory feedback in neuromuscular control of finely adjusted cranial systems.

## Graphical Abstract

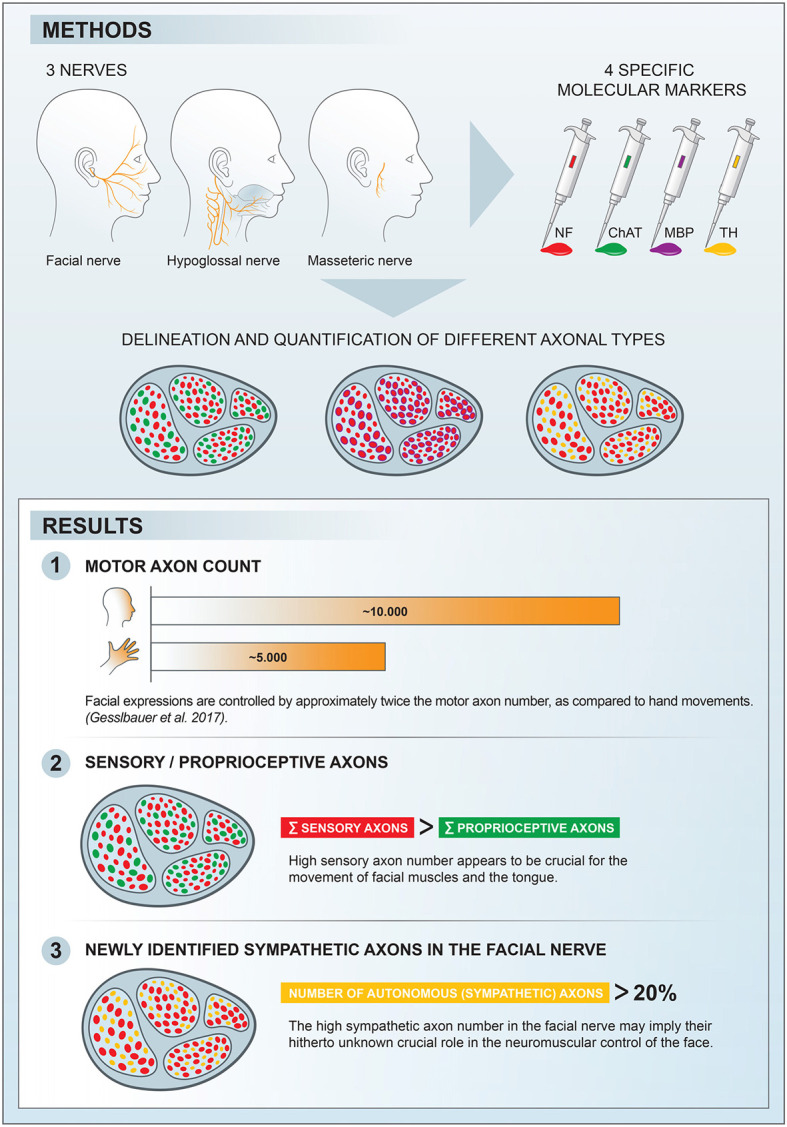

## 1. Introduction

Facial expression is a primary characteristic of human interactions. Overall, more than 40 facial mimic muscles generate about 10,000 facial nuances, as the result of highly sophisticated control mechanisms involving the limbic system, pre-frontal cortex, and medullary centers (Wehrle et al., 2000; Schmidt and Cohn, 2001; Burrows, 2008). Together with the masticatory and tongue muscles, the human cranial muscles are capable of ingestion of food, mastication, and speech in its various forms. Proprioceptive feedback is believed to enable multi-vectorial motion of the tongue, together with coordinated mastication movements (Lazarov, 2007). This is accomplished in a closed loop with the proprioceptive organs (i.e., muscle spindles) within the muscle bodies (Zapata and Torrealba, 1988; Scutter and Türker, 2001).

While masticatory and intrinsic tongue muscles are governed by nerves with a mixed neuronal population, containing motor and proprioceptive sources, the extracranial facial nerve has been long considered purely constituted by motor nerve fibers. Therefore, it has been historically accepted that control of the facial muscles is provided solely by motor neuronal input of the facial nerve and controversies regarding the nature of neural feedback from the facial muscles have emerged over the past years (Carmichael and Woollard, 1933; Cobo et al., 2017). The view of a purely motor facial nerve has been questioned in a recent experimental study (Tereshenko et al., 2023a) but the extent of non-motor axons in facial muscle control remains unclear yet.

In this study, we used molecular markers to identify different neuronal components at the axonal level of the facial nerve branches as well as the hypoglossal and masseteric nerves. The aim was to perform a distinctive axonal mapping of the various subtypes within the cranial nerves and to compare them with the peripheral nerves of the upper extremity from a previous study; thus, to gain insights into the control mechanisms of these complex neuromuscular systems (Gesslbauer et al., 2017). The secondary goal was to establish intraneural cartography and to identify functional pathways corresponding to the axon populations found within the studied nerves.

## 2. Materials and methods

### 2.1. Sample harvesting

Nerve samples were harvested from six human organ donors (age rage [43–101], 50% female) unilaterally immediately after death (<10 h postmortem). Organ donors were used to preventing the decay of the choline acetyltransferase (ChAT) and to ensure reliable signals across all nerve samples (Fahn and Côté, 1976; Pahud, 1998). Organ donors with facial palsy, neurodegenerative diseases, parotidectomy and facial trauma were excluded from the study. The facial nerve branches (main trunk of the extracranial facial nerve, temporal, zygomatic, buccal, mandibular, and cervical branches) as well as the masseteric and the hypoglossal nerves were harvested for immunofluorescence staining and axon quantification. Approval was obtained from the ethics committee of the Medical University of Vienna (reference number EK Nr: 1213/2012).

**FIGURE 1:**
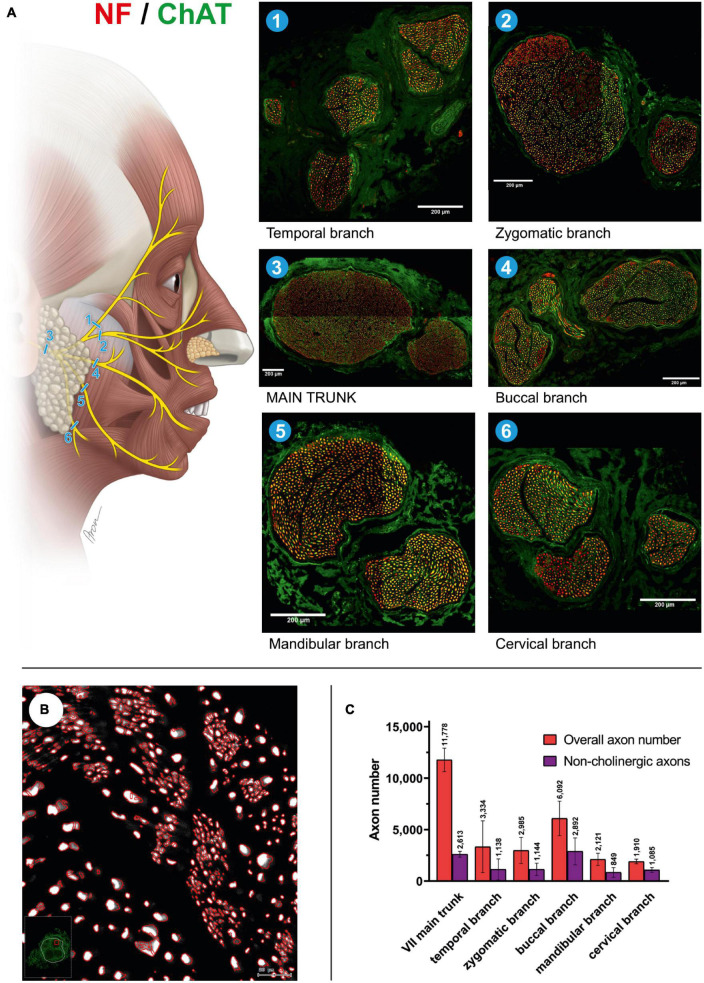
Mixed axonal populations of the facial nerve branches. **(A)** Schematic illustration of the facial nerve branches and corresponding cross-sections. The specimen is stained using anti-NF (red) and anti-ChAT (green) antibodies. Scale: 200 μm. **(B)** Semi-automated quantification analysis of axons in the cross-section of the mandibular branch of the facial nerve. NF-positive signals are automatically identified using the StrataQuest software (TissueGnostics, Vienna, Austria). **(C)** Overall and non-cholinergic axon numbers of the facial nerve branches are depicted. Data are presented as mean ± SD.

**FIGURE 2:**
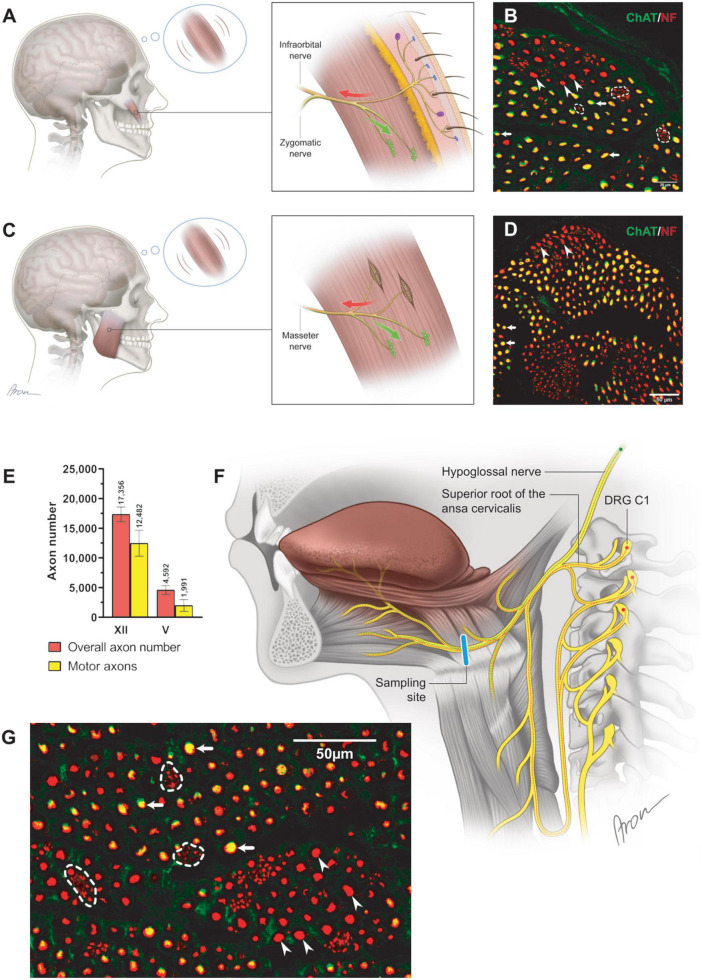
Neuromuscular control via the cranial motor nerves. **(A)** The schematically depicted zygomaticus major muscle is incorporated into the SMAS layer with the overlying skin (Happak et al., 1994; Gothard, 2014; Müri, 2016). Subtle deviations of the skin mirror dynamic position changes of the zygomaticus major muscle. The proximal part of the zygomatic nerve contains motor neuronal sources responsible for the innervation of the neuromuscular junctions (green) within the muscle. Distally, the motor zygomatic nerve merges with the sensory infraorbital nerve (V2), establishing a nerve with mixed neuronal composition. The sensory neuronal population (red) of the mixed nerve extends toward the facial skin by piercing the zygomaticus major muscle according to a previous study (Bankhead et al., 2017). The finest deviations of the skin are registered by the mechanoreceptors and transmitted via the infraorbital nerve to the CNS. Centrally processed signals allow for motor control adjustment of the zygomaticus major muscle via the zygomatic nerve. **(B)** Cross-section of the zygomatic nerve. The motor axons (arrows; yellow) are identified along clusters of non-myelinated fibers (dashed line). Additionally, thick, myelinated, non-cholinergic fibers (arrowheads) were identified suggesting their proprioceptive nature. **(C)** Closed-loop concept for neural feedback mechanism in the masseteric muscle. The masseteric nerve itself contains a mixed neuronal population **(D)**, contributing to motor innervation of the neuromuscular junctions (green) as well as sensory innervation (red) of the muscle spindles (Scutter and Türker, 2001). Information of the dynamic position changes of the masseteric muscle is recorded by the incorporated proprioceptive organs (muscle spindles), which are transmitted to the CNS via the same masseteric nerve. In contrast to the muscles of facial expression, motor control adjustments of the masseteric muscle rely on the own incorporated mechanosensitive sensors. Thus, finely tuned control of the diverse facial expressions may rely on the cutaneous sensory feedback from the facial skin. **(D)** In the magnified cross-section [×60] of the masseteric nerve, motor (cholinergic, ChAT-positive) fibers were predominant (arrows) while thick, non-cholinergic, myelinated fibers (arrowheads) represent the proprioceptive axonal population. **(E)** The proportion of cholinergic axons was 72 (12)% in the hypoglossal (*n* = 6) and 44 (19)% in the masseteric nerve (*n* = 4). Data are presented as mean ± SD. **(F)** The hypoglossal nerve emerges from the cranium as a motor nerve containing myelinated cholinergic (green) axons (Morecraft et al., 2001). Caudally, the hypoglossal nerve joins the superior root of the ansa cervicalis, which contains along with motor axons to the suprahyoid muscles’ thick afferent nerve fibers. Thus, proprioceptive axons (red) travel from the dorsal root ganglion (C1) via the ansa cervicalis to join the motor hypoglossal nerve. Distally to the ansa cervicalis the hypoglossal nerve represents a mixed nerve (red and green), containing proprioceptive and motor nerve fibers for innervation of the intrinsic tongue muscles. **(G)** In the magnified image [×60] of the hypoglossal nerve, motor (cholinergic, ChAT-positive) fibers were predominant (arrows) while thin axons gathered into conglomerates represent sympathetic fibers (dashed line). Thick, non-cholinergic, and myelinated fibers (arrowheads) represent the proprioceptive axonal population.

All nerves were exposed by two skin incisions. A preauricular incision was extended cranially beyond the hairline and was continued dorsocranially toward the hairline at the root of pinna. After dissecting the whole skin flap to the nasolabial fold, the main trunk of the extracranial facial nerve was exposed. Distal branches were identified by following the main trunk of the facial nerve toward the nasolabial fold. The harvesting sites were defined as 0.5 cm rostral from the anterior border of the parotid gland (Figure 1A). Due to the different branching patterns of the facial nerve branches, the authors dissected all distal branches first and then proceeded to identification and harvesting of the corresponding branches (Angelov, 2016). The motor branch to the masseter muscle was identified as 3 cm anterior to the tragus and 1 cm inferior to the caudal edge of the zygomatic arch (Borschel et al., 2012). The harvesting site for the hypoglossal nerve was determined based on clinical relevance as this nerve is commonly used in nerve reconstruction for facial reanimation. Therefore, via a submandibular incision the hypoglossal nerve was exposed and harvested inferior and medial to the posterior belly of the digastric muscle after emergence through the carotid artery bifurcation (see Figure 2F).

Harvested samples were immediately fixated by immersion in 4% paraformaldehyde (PFA) diluted in 0.1 M phosphate-buffered saline (PBS), pH 7.4, at +4°C, for 12–24 h. Afterward, the samples were extensively washed with PBS, pH 7.4, followed by dehydration in increasing sucrose/PBS solutions (10, 25, and 40%) for 24 h in each at +4°C. Afterward, the samples were embedded in Tissue-Tek^®^ O.C.T. tm Compound (Sakura Finetek Europe B.V., Alphen aan den Rijn, Netherlands) and stored at −80°C. Nerves were cut into 10 μm thick cross sections using a cryostat (Leica CM1950, Wetzlar, Germany).

### 2.2. Immunofluorescence labeling

The double immunofluorescence staining using anti-neurofilament (NF) and anti-choline acetyltransferase (ChAT) to distinguish cholinergic from non-cholinergic axons was performed as previously established and described (Gesslbauer et al., 2017; Tereshenko et al., 2023b). Primary antibodies were obtained from Sigma-Aldrich (St. Louis, MO, USA). Antibodies from Sigma-Aldrich included chicken anti-NF (catalog number AB5539; lot numbers: 3128840, 11212161), goat anti-ChAT (catalog number AB144P, lot numbers: 2780618, 3251012, 2079751), and rabbit anti-TH (catalog number: AB152, lot number: 390204) and rat anti-MBP (myelin basic protein [catalog number MAB386]). Anti-NF was used at concentration of 1:2000, anti-ChAT at 1:100, anti-TH at 1:250, and anti-MBP at 1:500. Secondary antibodies conjugated with Alexa Fluor 488 or 568 were obtained from Thermo Fisher (Waltham, MA, USA). All secondary antibodies were used at concentration of 1:500. Anti-NF is a pan-neuronal marker and was used to visualize nerve fibers. Acetylcholine transferase is the synthesizing enzyme for the neurotransmitter acetylcholine and anti-ChAT visualizes cholinergic axons. Sympathetic nerve fibers were identified by anti-TH antibodies. Despite the fact that anti-TH specificity has been shown to extend beyond sympathetic nature of the neuronal entities (Brumovsky, 2016; Vyas et al., 2017; Tang and Pierchala, 2022), authors confirmed the neural origin of the sympathetic nerve fibers of the facial nerve in the superior cervical ganglion in a previous study (Tereshenko et al., 2023a). The myelinated fibers were specifically visualized by anti-MBP.

Cross sections of the nerve samples were stained with (1) anti-NF and anti-ChAT, (2) anti-NF and anti-TH and (3) anti-NF and anti-MBP. Before labeling, frozen sections were air dried followed by incubation in 10% normal goat serum or 10% rabbit serum (staining combination 1) in PBS containing 0.1% Triton for 1 h. Thereafter, sections were incubated for 48 h with the primary antibodies, washed with PBS, and incubated for 2 h with the secondary antibodies. Finally, the tissue was rinsed again in PBS and mounted in a fluorescence mounting medium (Dako, Carpinteria, CA 93013 USA). Images of nerve cross-sections were acquired using a fully integrated imaging system (TissueFAXS; TissueGnostics, Vienna, Austria).

### 2.3. Confocal imaging

Fluorescently immunolabeled nerve sections were analyzed with a confocal laser scanning microscope (CLSM, Olympus FV3000, Olympus Europa SE & Co. KG, Hamburg, Germany). A series of virtual CLSM sections of 1 μm thickness were cut through the structures of interest. Each section was photo-documented with a 1024 × 1024 pixel resolution and 3D projections were rendered using Image J software (National Institutes of Health [NIH], Bethesda, MA, USA). Double-colored images were generated using lasers with excitation wavelength 488 and 568 nm.

### 2.4. Quantification analysis

Automated quantification of axons within the nerves samples was performed using StrataQuest version 5.1.249 and TissueQuest version 4.0.1.0128 (TissueGnostics, Vienna, Austria) as described previously and validated by Gesslbauer et al. (2017). Per sample three cross-sections were selected for quantification analysis. The results were calculated using a custom-made script made specifically for this staining protocol (“Fibers_v3_16bit”). Axons were identified and quantified according to the following criteria. NF signals were used as the focus channel as this identifies all axons. The ChAT-positive axons were counted when overlapping with NF signals. All single positive as well as double positive axons were counted and visualized in the nerve cross section. Manual post-analysis correction of the falsely identified axons was applied to every single sample in alle three cross-sections. The variance between different cross-sections from the same sample remained under 3%.

For axonal quantification of the cross-sections stained using anti-NF and anti-MBP, QuPath version 0.3.0 was used (Bankhead et al., 2017). NF-positive axons were detected using the cell detection module. Subsequently, object classification via a single measurement classifier was used to classify MBP-positive axons by thresholding for mean MBP intensity in a 1 μm encircling each single axon.

### 2.5. Statistical analysis

Statistical analysis was not performed due to *de facto* descriptive and normative nature of this study of axon counts within the nerves studied. Descriptive statistics are presented for all nerve samples, and data are presented either as absolute and relative values as well as means and standard deviations.

## 3. Results

### 3.1. Mixed axonal composition of the extracranial facial nerve

**FIGURE 3:**
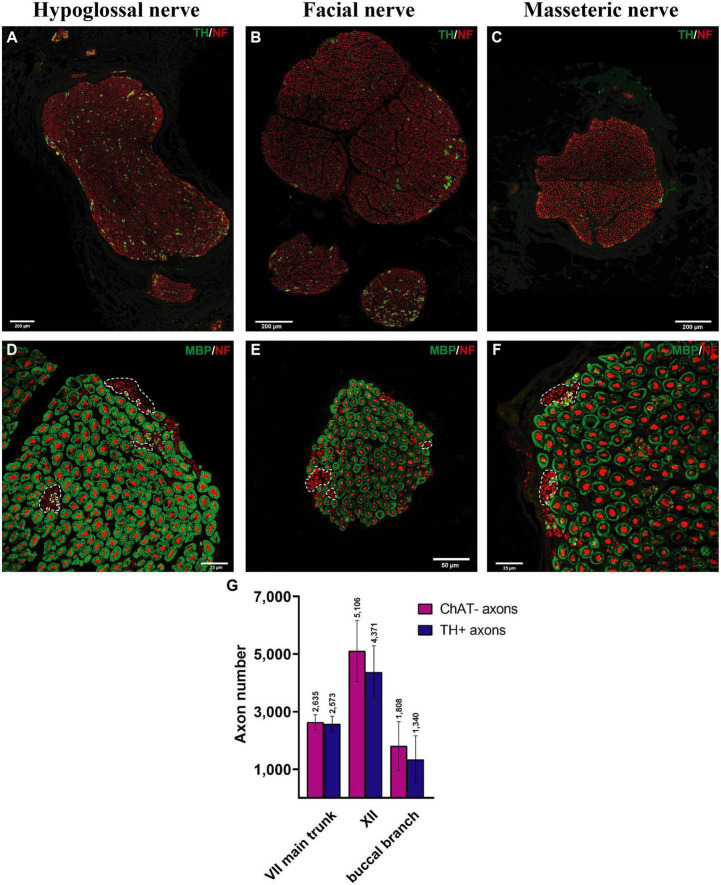
Myelinated and sympathetic axonal populations in the cranial motor nerves. Myelinated and sympathetic fibers of the hypoglossal **(A,D)**, facial **(B,E)**, and masseteric **(C,F)** nerves. Cross-sections of all nerves showed predominantly myelinated nerve fibers (MBP in green color; see **D–F**). Clusters of non-myelinated fibers were observed in all nerves (dashed line) and stained negative for MBP **(D–F)**, indicating their non-myelinated nature. These non-myelinated axons correspond with tyrosine hydroxylase positively stained axons in Figure 4. Quantification analysis revealed matched numbers of ChAT- and TH + axons **(G)**.

**FIGURE 4:**
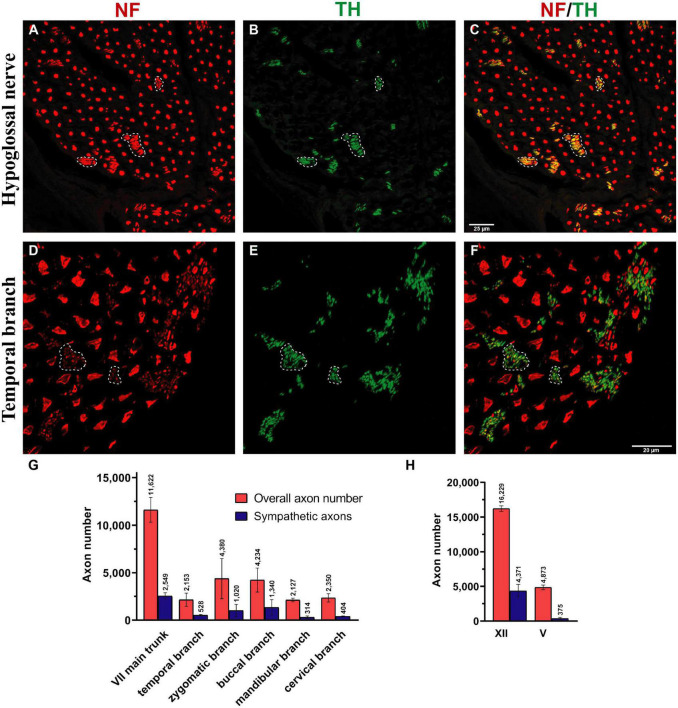
Sympathetic axonal population in the hypoglossal and facial nerve branches. Clusters of non-myelinated fibers were observed in all nerves (dashed line) and stained positive for tyrosine hydroxylase (TH in green color; see **B,E**) in hypoglossal and temporal nerves, indicating their sympathetic nature. **(A,D)** The entirety of axons was identified using a pan-neuronal neurofilament (NF) antibody (in red color). **(B,E)** The sympathetic axons were labeled with a TH antibody (in green color). **(C,F)** The overlay demonstrates a large area of NF-positive and TH-positive axons (dashed line), indicating the sympathetic nature of the smaller-caliber axons. **(G,H)** Quantification revealed a high proportion of sympathetic fibers in all facial nerve branches and the hypoglossal nerve: 15–30%. In comparison, the masseteric nerve only contained 7.6 (3)% sympathetic nerve fibers **(H)**. Data presented as mean ± SD.

The main trunk of the extracranial facial nerve demonstrated a mixed axonal population. The overall axon count in the main trunk of the facial nerve on one side comprised 12,800 (1,100) axons, whereby only 78 (2.3)% were motor axons (Table 1). Using antibodies against NF and ChAT, nerve samples from all organ donors demonstrated a proportion of ChAT-negative (non-cholinergic) fibers (Figures 1A, C). Thus, the non-cholinergic fibers represent a newly identified axonal population in the main trunk of the extracranial facial nerve. To specify the nature of these non-cholinergic axons, two additional stainings were performed. In the first one, we demonstrated that non-cholinergic fibers of the facial nerve were non-myelinated because they lacked myelin sheath (MBP-positive signals) immunoreactivity (Figure 3). Another staining showed that these non-cholinergic and non-myelinated fibers were gathered in clusters and showed a positive signal against tyrosine hydroxylase across the whole cross-section of the facial nerve, indicating their sympathetic origin and nature (Figure 4). The number of ChAT-negative axons matched with the number of TH-positive axons: 2,635 (264) vs. 2,573 (272) respectively (Figure 3G). This indicated two main axon populations in the main trunk of the facial nerve: motor and sympathetic axons with a relative relation of approximately 4:1.

**TABLE 1:** Axon quantification of the motor cranial nerves.

	Overall axon number	Motor axons (ChAT^+^)	Non-cholinergic axons (ChAT^–^)	% of motor axons
**Facial nerve with distal branches**				
Main trunk of the facial nerve (VII)	11,778 ± 1,138	9,165 ± 1,013	2,613 ± 280	77.7 ± 2.3%
Temporal branch	3,334 ± 2,518	2,197 ± 1,529	1,138 ± 1,022	67.9 ± 9.8%
Zygomatic branch	2,985 ± 1,269	1,842 ± 787	1,144 ± 600	61.3 ± 9.6%
Buccal branch	6,092 ± 1,672	3,200 ± 1,128	2,892 ± 1,305	53.0 ± 14.2%
Mandibular branch	2,121 ± 594	1,272 ± 334	849 ± 464	61.8 ± 13.3%
Cervical branch	1,910 ± 229	825 ± 231	1,085 ± 243	43.2 ± 10.0%
**XII and V cranial nerves**				
Hypoglossal nerve	17,356 ± 1,227	12,482 ± 2,171	4,875 ± 2,070	71.9 ± 11.6%
Masseteric nerve*^,1^	4,592 ± 740	1,991 ± 995	2,601 ± 1,131	43.6 ± 19.2%

Data are shown as mean ± standard deviation, *n* = 6.

*The descending branch of the masseteric nerve was harvested and analyzed.

^1^*n* = 4.

### 3.2. Afferent axon population of facial nerve branches

**FIGURE 5:**
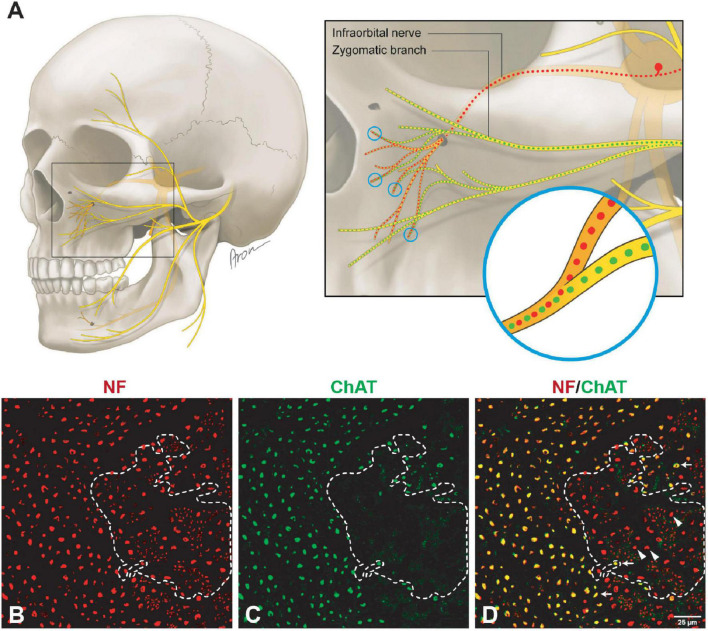
Mixed axonal composition of the trigeminal-facial interconnections. **(A)** Schematic illustration of the trigeminal-facial interconnections containing a mixed axonal population (Bankhead et al., 2017). The zygomatic branch emerges from the main trunk of the facial nerve as a motor nerve containing myelinated cholinergic fibers (green). Distally, facial nerve branches merge with the trigeminal nerve branches (infraorbital nerve depicted). Non-cholinergic axons (suggesting their afferent nature) travel from the trigeminal ganglion via the infraorbital nerve to merge with facial nerve branches building a mixed trigeminal-facial nerve. **(B–D)** Different axon types within the zygomatic branch (60× magnification). **(B)** The entirety of axons was identified using a pan-neuronal neurofilament (NF) antibody (in red color). **(C)** The motor (cholinergic) fibers were labeled with a choline acetyltransferase (ChAT) antibody (in green color). **(D)** The overlay demonstrates a large area of NF-positive and ChAT-negative axons (dashed line), indicating the non-cholinergic nature of the smaller axons. The NF- and ChAT-positive axons are cholinergic motor axons (arrows). The thicker non-cholinergic fibers are suggestive of afferent axons (arrowheads).

Unlike the main trunk of the facial nerve, distal facial nerve branches contained an additional population of myelinated afferent fibers (ChAT-negative), along with motor and sympathetic axons (Figures 1A, 5). All nerves branching off the main facial nerve trunk showed afferent fibers in the analyzed cross sections. Quantitative analysis showed that the number of myelinated nerve fibers (MBP-positive) was higher compared to motor (ChAT-positive) fibers (e.g., in the temporal branch: 1,192 vs. 1,074; in the buccal branch: 3,984 vs. 3,851). Moreover, the number of non-cholinergic (ChAT-negative) axons was higher than the number of sympathetic (TH-positive) axons in the buccal branch: 1,808 (850) vs. 1,340 (822). This indicated the presence of three different axon populations of the distal facial nerve branches: motor, sympathetic, and afferent.

### 3.3. Motor and sympathetic fibers of the facial nerve branches

The individual facial nerve branches demonstrated a mixed axonal composition as well (Figure 5C). The motor axon proportion was 68 (9.8)% in the temporal, 61 (9.6)% in the zygomatic, 53 (14)% in the buccal, 62 (13)% in the mandibular, and 43 (10)% in the cervical branches (Table 1). All non-myelinated, non-cholinergic axons of the facial nerve branches are of sympathetic nature due to their positive signal against tyrosine hydroxylase (Figures 4F, G). The respective portion of the sympathetic fibers in facial nerve branches was 25 (4.4)% in the temporal branch, 22 (3.9)% in the zygomatic branch, 30 (10)% in the buccal branch, 15 (4.5)% in the mandibular branch and 18 (4.9)% in the cervical branch (Figure 4G and Table 1). The sympathetic fibers formed conglomerates diffusely spread across the nerves’ cross sections (Figures 4C, F).

### 3.4. Mixed axon population in the hypoglossal and masseteric nerves

Both hypoglossal and masseteric nerves showed a mixed axonal population as well. The motor branch to the masseter muscle showed an overall count of 2,000 (995) [44 (19)%] motor axons (Figures 2D, G and Supplementary Figure 1). The masseteric nerve showed a non-myelinated axonal population, which is of sympathetic nature, comprising 7.6 (2.8)% of the overall axonal population (Figure 3C and Supplementary Figure 1). Furthermore, the masseteric nerve also showed a myelinated afferent axon population (ChAT-negative), indicating proprioceptive function (Figures 2, 3 and Supplementary Figure 2).

The monofascicular part of the hypoglossal nerve consisted of overall 17,400 (1,200) axons with 72 (12)% motor axons. The sympathetic axon proportion was 27 (6.4)% (Figures 3, 4H and Supplementary Figure 2). Similar to distal facial nerve branches and the masseteric nerve, the hypoglossal nerve contained myelinated non-cholinergic fibers (Figure 2). The number of non-cholinergic (ChAT-negative) axons was higher than the number of sympathetic (TH-positive) axons in the hypoglossal nerve: 5,106 (1,058) vs. 4,371 (923) (Figure 3G), indicating the hypoglossal nerve’s proprioceptive properties for the intrinsic tongue muscles.

## 4. Discussion

Facial expressions, a fundamental aspect of human interaction, can be produced in a variety of about 10,000 nuances by activation of the mimic muscles controlled by the limbic system, prefrontal cortex, and vital medullar centers (Gothard, 2014; Müri, 2016). Aside from the complexity of central neuronal control, the peripheral morphologic correlates for proprioceptive feedback in the facial muscles are not clearly evident (Happak et al., 1994; Morecraft et al., 2001; Cobo et al., 2017). Moreover, dysfunctions of the facial neuromuscular system often require restoration of natural motor control, which represents a great clinical challenge. This study demonstrates mixed axonal populations (motor, sensory, and sympathetic) of the facial, hypoglossal and masseteric nerves. These different neuronal populations of the nerves responsible for tongue movement, mastication, and facial expressions shed light on the control mechanisms of these complex neuromuscular systems (Figure 2). Contribution of the afferent neuronal population via the trigeminal-facial interconnections suggest the presence of afferent feedback from the facial muscles. The serendipitous finding on the abundance of sympathetic axons in the motor cranial nerves suggests an involvement of the autonomic nervous system in involuntary control of the muscle tone in the facial muscles, which is indispensable for non-verbal human interactions.

Our findings showed approximately twice the overall axon number for the main trunk of the facial nerve compared to other studies: 11,778 ± 1,138 vs. 6,254 (range 4,486–7,570) by Fujii and Goto (1989), 6,684 ± 1,884 by Engelmann et al. (2020), and 5,329 ± 1,376 by Hembd et al. (2017). The axon number discrepancies are most likely explained by the use of different staining techniques in other studies, which in turn only allowed for the identification of myelinated nerve fibers (Captier et al., 2005). Our double immunofluorescence technique provided absolute overall axon numbers along with the specification of axonal types based on distinct molecular markers. Most importantly, our findings indicate accurate numbers of cholinergic axons in all facial nerve branches, which have not been reported so far (Table 1). Moreover, we provided molecular evidence of the afferent axon population of the distal facial nerve branches, i.e., myelinated non-cholinergic sensory nerve fibers, which elucidates the elusive proprioceptive control of facial muscles (Figure 5).

While the proprioceptive perception of tongue movement is well-understood, evidence for proprioception in the facial muscle system is poorly explored. According to our findings from an animal model, proprioceptive signals are not conducted via the facial nerve (Tereshenko et al., 2023a). Skeletal muscles of the lower and upper extremities are known to provide proprioceptive feedback due to the incorporated muscle spindles. In the upper extremity, sensory and motor axon populations emerge separately as dorsal and ventral roots from the spinal cord, and fuse right at the intervertebral canal, forming a mixed spinal sensorimotor nerve. This proximal fusion of sensory and motor neural sources implies a mixed axonal nature of all nerve branches to muscles in the extremities (Gesslbauer et al., 2017). On the other hand, the nuchal muscles (e.g., sternocleidomastoid muscle) or intrinsic tongue muscles are controlled by motor neural sources via accessory and hypoglossal nerves respectively, which to date were considered “pure” cranial motor nerves. Nevertheless, both nuchal and lingual systems provide potent proprioceptive feedback, which is located in cervical dorsal root ganglia and is transmitted via “sensory” nerve branches [cervical plexus for nuchal musculature and ansa cervicalis for the tongue, (Figure 2F)] (Cooper, 1953; Zapata and Torrealba, 1988; Zenker et al., 1988; O’Reilly and FitzGerald, 1990). This indicates that the fusion of motor and sensory neural sources in these cranial neuromuscular systems occurs more distally in direct proximity to the muscle. The sensorimotor integration of the facial-trigeminal system at the subcortical level is well studied (Nguyen and Kleinfeld, 2005). The trigeminal-facial loop is closed by direct projection from the trigeminal nuclear complex to the facial nucleus and indirect pathways within the brainstem via the pontomedullary reticular formation (Zerari-Mailly et al., 2001). Recent studies focused on the proprioceptive feedback at the muscular level, enlightening elusive proprioceptive entities within the facial muscles (Cobo et al., 2017). This notion raises an intriguing question of whether the trigeminal-facial interconnections represent a mixed neural population, which is responsible for both motor and proprioceptive innervation of the facial muscles (Baumel, 1974; Diamond et al., 2011; Hwang et al., 2015). While our findings showed an additional afferent axonal population in the distal facial nerve branches in contrast to the proximal main trunk of the facial nerve, there is no explicit evidence that sensory input occurs via the trigeminal nerve (Dixon, 1899; Davis, 1923; Davis et al., 1956; Figures 1, 5). However, these myelinated afferent fibers in facial nerve branches may be of proprioceptive nature, which travel via trigeminal branches to central nuclei corresponding to trigeminal nerve function. Hence, the trigeminal branches seem to complement these distal branches with afferent fibers to provide the facial muscles with a mixed axon population. Thus, this anatomic interplay, along with recent evidence of newly identified mechanosensitive corpuscles within the facial muscular system, may explain the nature of proprioceptive feedback from the facial muscles.

Another finding of this study is the presence of sympathetic axons within facial, masseteric, and hypoglossal nerves. While autonomic nerve fibers travel within sensory branches of the trigeminal nerve to the blood vessels and sweat glands, the role of sympathetic fibers in the motor cranial nervous system is poorly described (Siemionow et al., 2011). However, recent studies indicated the involvement of sympathetic nerve fibers in the modulation of neuromuscular junctions and various effects on the neuromuscular homeostasis (Khan et al., 2016; Rodrigues et al., 2019). Although the physiological role of sympathetic axons in the cranial neuromuscular system remains unclear, we have shown that they are abundant within the cranial motor nerves studied (Figures 3, 4). A recent study suggests presence of multiple bundles of noradrenergic unmyelinated axons within the abducens nerve (Mansour and Kulesza, 2023). In an experimental study by Shibamori et al. (2004) no tyrosine hydroxylase-positive axons were observed beyond the stylomastoid foramen (i.e., in the extracranial facial nerve) in the rat. However, our previous findings demonstrated the presence of sympathetic axons in the extracranial facial nerve in the rat. Only 2.4% of sympathetic axons were identified in the main trunk of the facial nerve in the rat [vs. over 20% in humans (Figure 4)], which were traced to the facial muscles (Tereshenko et al., 2023a). The neural route of the sympathetic axons in the facial nerve was demonstrated to travel via the facial and not the trigeminal nerve by selective denervation of the trigeminal nerve branches in the rat model (Tereshenko et al., 2023a). Sympathetic fibers may travel from the superior cervical ganglion through the adventitia of a vessel of the posterior circulation to the extracranial facial nerve (Tereshenko et al., 2023a). The higher proportion of the sympathetic axons in the human facial nerve can represent a fundamental evolutionary adaptation, which has arisen as a result of emotional expressions. Sympathetic activity may play an essential role in maintaining muscle tone of the facial muscles without the involvement of the CNS, to ensure continuous facial expressions at a basic level needed for interhuman facial recognition and social interactions (Rodrigues et al., 2019). However, this hypothesis may contradict the clinical manifestation of Horner’s syndrome with no reported impairment of facial expressions despite disturbance of the sympathetic activity (Kanagalingam and Miller, 2015). Moreover, sympathetic nerve fibers may be involved in different pathological conditions associated with involuntary facial muscle contractions like a hemifacial spasm or postoperative synkinesis after facial nerve reconstruction (Zheng et al., 2012; Dou et al., 2015). Thus, the higher sympathetic axonal population in the facial nerve, compared to the hypoglossal and the lowest in the masseteric nerves may explain why the using facial nerve branches as a donor nerve provides more muscle tone after reinnervation compared to other donor nerves (Eisenhardt et al., 2013). The sympathetic contribution may play an essential role in the pathophysiology behind postoperative synkinesis as well, which requires further investigation.

The cranial nerves analyzed in this study were shown to possess much higher numbers of motor axons compared to the terminal nerves, which originate from the brachial plexus to innervate upper limb muscles (Gesslbauer et al., 2017). According to recent findings, finely tuned movements of the hand is controlled by less than 5,000 motor neurons (Gesslbauer et al., 2017). Interestingly, the accurate motor control of dexterous hand movements seems to depend much more on indispensable sensory feedback, since all terminal nerves innervating the human arm were found to consist of more than 80% sensory axons. Facial and tongue movements are controlled by neuromuscular systems exhibiting even higher complexity and interrelations. The neural drive to muscles in the face is transmitted by motor axons of a twice greater number than needed for controlling hand and finger movements (Figure 2). This indicates a higher innervation ratio of cranial motor neurons, whereby one motor neuron is responsible for the control of fewer muscle fibers, hence, establishing smaller motor units (Sawczuk and Mosier, 2001). This notion may highlight the necessity of high-precision neural control of a single muscle to achieve the finest facial movements.

High motor unit number in the facial muscles highlights high-resolution control, however, does not explain how sensory feedback is processed in cranial neuromuscular systems. The origin of neural feedback in intrinsic tongue control may be of an exteroceptive nature (Trulsson and Essick, 1997; Saigusa et al., 2006). The tongue as a sensory organ can sense distinctive sensory modalities via high-fidelity gustatory and somatosensory receptors of the tongue’s surface area and transmits them via sensory lingual and glossopharyngeal nerves to the central nervous system (Mu and Sanders, 2010). Moreover, less versatile movement of the masticatory muscles has been shown to depend largely on intraoral afferent signals and not on proprioceptive input from the masticatory musculature (Trulsson and Johansson, 2002). These reports, along with our findings, suggest that exteroceptive feedback is superior to proprioceptive feedback regarding coordinated and finely tuned motor control of cranial neuromuscular systems. This can apply to the human facial dermato-muscular system as well since it represents an evolutionary remnant of a somatosensory whisker system in mammals (Huber, 1930). Thus, the sensory feedback of the facial neuromuscular system may be mediated via facial cutaneous sensory organs, which detect small deviations of the skin and, therefore, modulate facial muscle contractions accordingly. However, it is still unclear whether the afferent neuronal population of the facial nerve branches originates from cutaneous mechanoreceptors or from mechanosensitive organs within the facial muscles (Cobo et al., 2017).

## 5. Conclusion

While the findings on the mixed axonal populations in the cranial nerves represent an intriguing phenomenon, the interpretation of the data and direct translation onto possible clinical implications is limited. To be able to support the conclusions of this study, further electrophysiological and experimental investigations are needed. Nevertheless, the findings emphasize the unique nature of motor control from highly complex cranial neuromuscular systems exhibiting finely tuned facial expressions and versatile vocal communications. On one hand, a higher number of motor axons innervate low muscle volume, compared to muscles in the upper extremity (Gesslbauer et al., 2017); on the other hand, rich sensory feedback transmitted from mechanoreceptors seems to play a pivotal role in the dexterous movement of the human facial and intrinsic tongue muscles. These differences imply a very different approach to closed-loop control of finely tuned cranial neuromuscular systems.

## Data availability statement

The original contributions presented in this study are included in the article/Supplementary material, further inquiries can be directed to the corresponding author.

## Ethics statement

The studies involving human participants were reviewed and approved by the Ethics Committee of the Medical University of Vienna (reference number EK Nr: 1213/2012). Written informed consent for participation was not required for this study in accordance with the national legislation and the institutional requirements.

## Author contributions

VT, UM, LH, DF, RB, KB, and OA: conception and design. VT, UM, DD, GL, LH, and OA: sample harvesting. VT, UM, GC, OP, LH, RB, and OA: imaging analysis. VT, UM, GL, OP, LH, DF, RB, KB, and OA: analyses and interpretation of data. DF, RB, KB, and OA: supervision. VT, CF, AG, DF, RB, and OA: drafting of the manuscript. All authors contributed to the critical revision and final approval of the version to be published.
