# Supplementary material for: Axonal mapping of the motor cranial nerves
Source: Front Neuroanat. 2023 Jun 2;17:1198042. doi: 10.3389/fnana.2023.1198042 (PMC10272770; doi:10.3389/fnana.2023.1198042)
Supplement: Supplementary file 1 [file Data_Sheet_1.PDF]

Title

Axonal mapping of the motor cranial nerves

Authors

Vlad Tereshenko[1,2], Udo Maierhofer[1], Dominik C. Dotzauer[1], Gregor Laengle[1], Olga Politikou[1], Genova Carrero Rojas[3], Christopher Festin[1,2], Matthias Luft[1,4], Florian J. Jaklin[1], Laura A. Hruby[1,5], Andreas Gohritz[6], Dario Farina[7], Roland Blumer[3], Konstantin D. Bergmeister[1,4], Oskar C. Aszmann[1,8*]

Affiliations

[1]Clinical Laboratory for Bionic Extremity Reconstruction, Department of Plastic, Reconstructive and Aesthetic Surgery, Medical University of Vienna, Vienna 1090, Austria

[2]Division of Plastic and Reconstructive Surgery, Massachusetts General Hospital, Harvard Medical School, Boston, MA, USA

[3]Center for Anatomy and Cell Biology, Medical University of Vienna, Vienna 1090, Austria

[4]Karl Landsteiner University of Health Sciences, Department of Plastic, Aesthetic and Reconstructive Surgery, University Hospital St. Poelten, Krems 3100, Austria

[5]Department of Orthopedics and Trauma Surgery, Medical University of Vienna, Vienna 1090, Austria

[6]Department of Plastic Surgery, University of Basel, Basel 4001, Switzerland

[7]Department of Bioengineering, Imperial College London, London SW7 2BX, UK

[8]Department of Plastic, Reconstructive and Aesthetic Surgery, Medical University of Vienna, Vienna 1090, Austria

Corresponding author: Oskar C. Aszmann*
oskar.aszmann@meduniwien.ac.at

Supplementary information

Figure S1 is related to Figure 3.

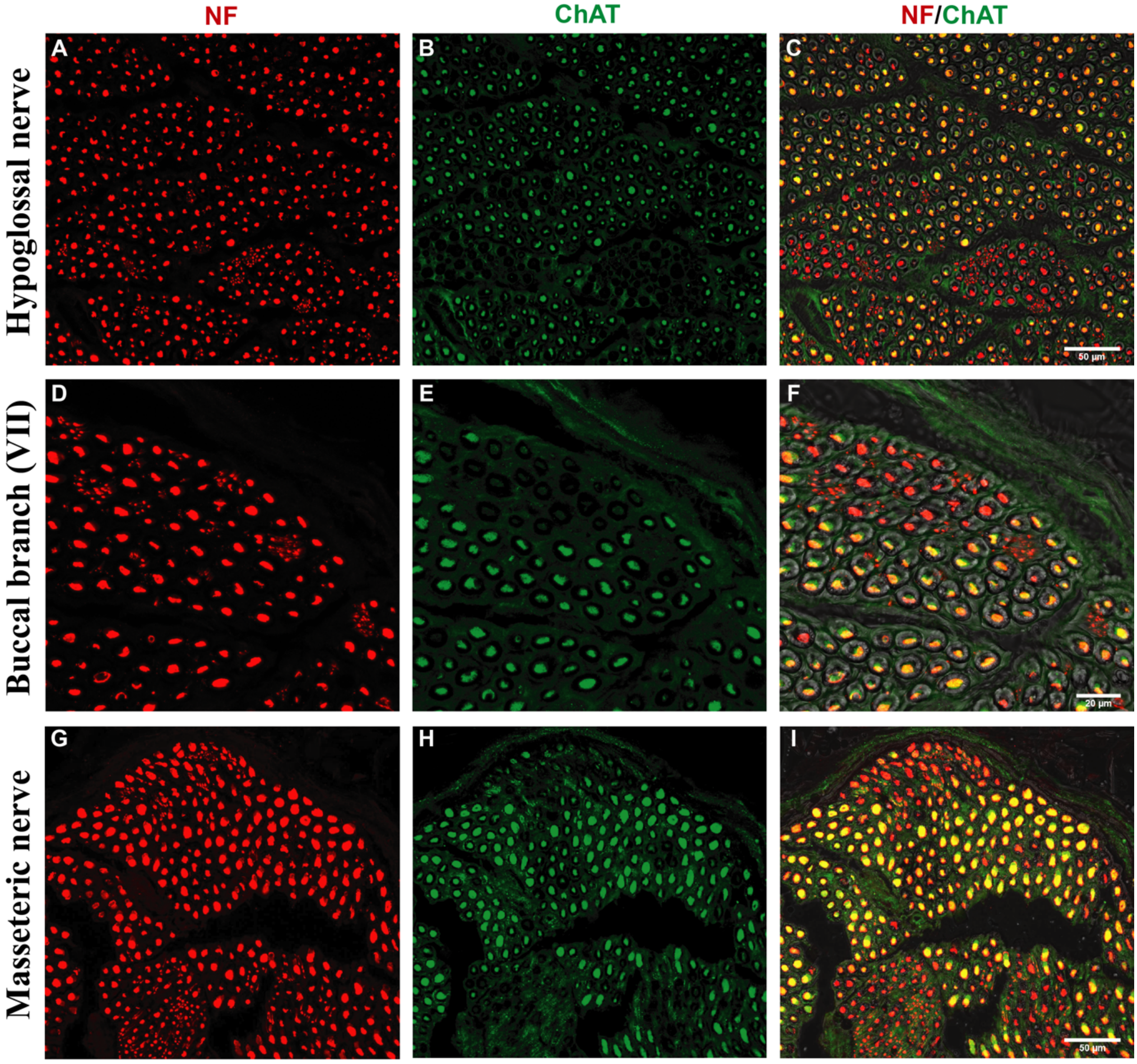

Fig. S1. Multi-channel images of the cranial nerves. *(A, D, G)The entirety of axons was identified using a pan-neuronal neurofilament (NF) antibody (in red color). (B, E, H) The motor (cholinergic) fibers were labeled with a choline acetyltransferase (ChAT) antibody (in green color). (C, F, I) The overlay demonstrates a large area of NF-positive and ChAT-negative axons, indicating the non-cholinergic nature of the smaller axons. The NF- and ChAT-positive axons are cholinergic motor axons. The thicker non-cholinergic fibers are suggestive of afferent axons.*

Figure S2 is related to Figure 4.

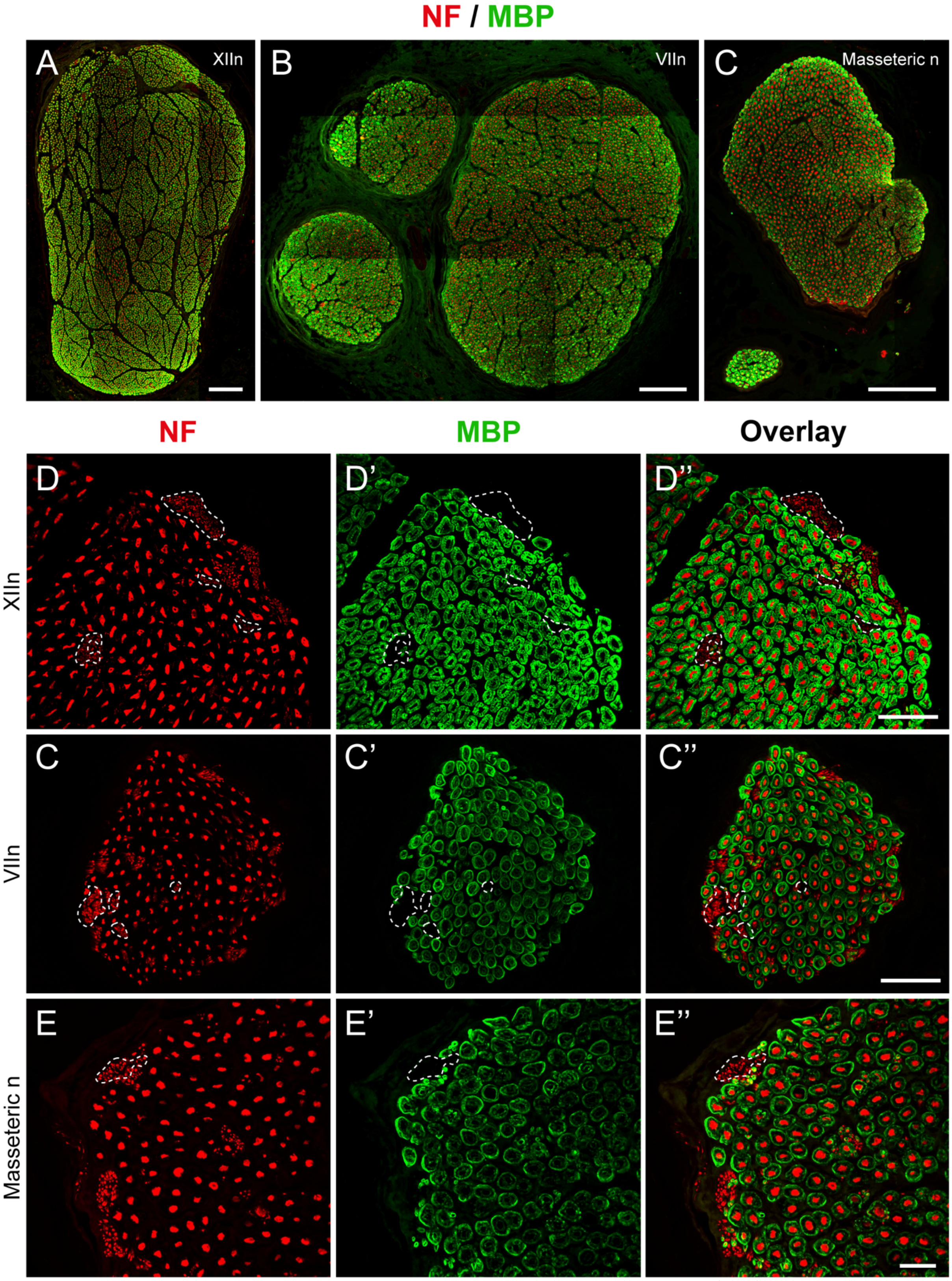

Fig. S2. Mixed axonal populations of the facial nerve branches. *(A) Schematic illustration of the facial nerve branches and corresponding cross-sections. The specimen is stained using anti-NF (red) and anti-ChAT (green) antibodies*. Scale: 200 μm*. (B) Semi-automated quantification analysis of axons in the cross-section of the mandibular branch of the facial nerve. NF-positive signals are automatically identified using the StrataQuest software (TissueGnostics, Vienna, Austria). (C) Overall and non-cholinergic axon numbers of the facial nerve branches are depicted. Data are presented as mean±SD.*
